# One Year of Enzyme Replacement Therapy Reduces Globotriaosylceramide Inclusions in Podocytes in Male Adult Patients with Fabry Disease

**DOI:** 10.1371/journal.pone.0152812

**Published:** 2016-04-15

**Authors:** Behzad Najafian, Camilla Tøndel, Einar Svarstad, Alexey Sokolovkiy, Kelly Smith, Michael Mauer

**Affiliations:** 1 Department of Pathology, University of Washington, Seattle, United States of America; 2 Department of Clinical Medicine, University of Bergen, Bergen, Norway; 3 Department of Pediatrics, Haukeland University Hospital, Bergen, Norway; 4 Department of Medicine, Haukeland University Hospital, Bergen, Norway; 5 Departments of Pediatrics and Medicine, University of Minnesota, Minneapolis, United States of America; Baylor Research Institute, UNITED STATES

## Abstract

Fabry nephropathy is associated with progressive accumulation of globotriaosylceramide (GL3) in podocytes. Reducing this GL3 burden may reduce podocyte injury. Sensitive methods to quantify podocyte GL3 content may determine whether a given strategy can benefit podocytes in Fabry disease. We developed an unbiased electron microscopic stereological method to estimate the average volume of podocytes and their GL3 inclusions in 6 paired pre- and post-enzyme replacement therapy (ERT) biopsies from 5 men with Fabry disease. Podocyte GL3 content was regularly reduced (average 73%) after 11–12 months of ERT. This was not detectable using a semi-quantitative approach. Parallel to GL3 reduction, podocytes became remarkably smaller (average 63%). These reductions in podocyte GL3 content or size were not significantly correlated with changes in foot process width (FPW). However, FPW after ERT was significantly correlated with the magnitude of the decrease in podocyte GL3 content from baseline to 11–12 months of ERT. Also podocytes exocytosed GL3 inclusions, a phenomenon correlated with their reduction in their GL3 content. Demonstrable after11–12 months, reduction in podocyte GL3 content allows for early assessment of treatment efficacy and shorter clinical trials in Fabry disease.

## Introduction

Deficiency of α-galactosidase A (αGal-A) in Fabry disease leads to the accumulation of its substrates, mainly globotriaosylceramide (GL3) in various cell types and organs [1], often eventuating in severe complications including strokes, cardiomyopathy, arrhythmias, neuropathy, renal failure and premature death [2].

While enzyme replacement therapy (ERT) eliminates visible GL3 accumulation in kidney endothelial and mesangial cells and fibroblasts within 5 months [3], podocytes, distal tubular cells and arteriolar smooth muscle cells are more resistant to ERT[3,4]. Although a long-term randomized placebo controlled ERT trial demonstrated reduced serious clinical events [5], there are substantial residual risks despite ERT [6]. Histological treatment responses have been important endpoints for clinical trials [3,7]. Studies of benefits of new treatments for patients already on ERT will need to focus on more resistant cells because endothelial GL3 clearance, the original criterion for ERT efficacy, will not be an available endpoint since GL3 clearance from these cells by ERT is essentially complete. Trials with hard endpoints would be of such long duration and size as to be impractical. Thus, the availability of structural endpoints that are sensitive early indicators of Fabry disease treatment efficacy and predictors of residual risk would be of substantial clinical significance.

**Fig 1:**
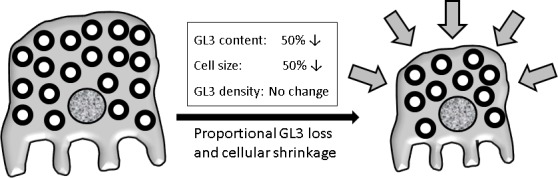
Schematic representation of how, due to a change in cell size, the "reference trap" may mask detection of intracellular GL3 reduction. While the podocyte on the left loses 50% of its GL3 content, because of a proportional (50%) shrinkage in podocyte cytoplasmic volume, the fraction of the volume of the podocyte cytoplasm filled with GL3 [Vv(Inc/PC)] remains the same (the cell on the right).

The podocyte is an important candidate in this regard. There is a dose-dependent benefit of 5 years of ERT on podocyte damage [8]. Critical for preventing urinary protein loss [9], podocyte depletion is implicated in glomerular scarring [10]. In young ERT-naïve Fabry disease patients the fraction of the volume of podocyte cytoplasm occupied by GL3 inclusions [Vv(Inc/PC)] increases with age, but not so in endothelial or mesangial cells [11]. Since the risk of Fabry disease complications is highly age-dependent [6,12], cells with no age-dependent progressive damage are less likely to contribute to progressive organ dysfunction. This has important implications for the selection of tissue treatment endpoints in clinical studies. Proteinuria is a strong Fabry nephropathy risk predictor. [6] Vv(Inc/PC) correlated with podocyte foot process width (FPW) [11], an indicator of podocyte injury [13], and both FPW and Vv(Inc/PC) correlated with urinary protein excretion in these young patients [11]. Although subjective light microscopic scoring systems detected reductions in podocyte GL3 after several years of ERT[4,8], earlier benefits are difficult to demonstrate using such methods. [3] To facilitate Fabry disease clinical trials of practical length and to allow earlier assessments of treatment efficacy, it is important to develop more sensitive assessments of changes in podocyte GL3. A scoring system for podocyte GL3 inclusions was developed by the International Study Group for Fabry Nephropathy (ISGFN). [14] Using a similar scoring system experienced observers could not detect increasing podocyte GL3 with increasing age [15,16] while morphometric measures of Vv(Inc/PC) using many of these same and a few additional biopsies elucidated robust correlations with age. [11] We posited that the failure to detect ERT benefits on podocyte GL3 could be methodological. If the total amount of GL3 per podocyte were diminished and, there was a parallel decrease in average podocyte volume, observers’ scores would not likely change as this method is insensitive to cell volume changes. This is termed ‘the reference trap’[16,17] whereby changes in the reference volume, *e*.*g*., mean podocyte volume could confound the estimate of per podocyte GL3 burden (Fig 1). Applying novel morphometric methods to test this hypothesis we determined that 11–12 months of ERT regularly decreased GL3 inclusion volume/podocyte [V(Inc/PC)], based entirely on decreased podocyte volume, since GL3 inclusions as a fraction of podocyte cytoplasmic volume [Vv(Inc/PC)] was unchanged.

## Materials and Methods

### Patients

Studies were performed in accordance with principles of the Declaration of Helsinki and were approved by the Institutional Review Board of the University of Minnesota, and the Regional Ethics Committee of Western Norway. Written informed consent had been obtained from each subject. Six males with Fabry disease [5 from the Fabrazyme^®^ phase III clinical trial [3]] treated with 1 mg/kg/EOH agalsidase-beta for 11 or 12 months were selected based on the availability of baseline and follow-up biopsies. Fabry disease was confirmed by measurement of leukocyte alpha-galactosidase A activity and/or GLA sequencing. Glomerular filtration rates were estimated based on the Modification of Diet in Renal Disease (MDRD) formula. [18]

### Kidney Biopsy

Baseline kidney biopsies prior to and after 11 or 12 months of ERT were performed as part of the clinical trial protocol or as a standard of care for assessment of severity of baseline Fabry nephropathy and ERT effectiveness. Biopsies from 9 healthy kidney donors provided normal control values. Biopsies were fixed in 2.5% glutaraldehyde and embedded in Poly/Bed®; 1 um sections were stained with toluidine blue for identification of glomeruli and scoring of GL3 inclusions in podocytes [14]. Random glomerular sections were prepared for stereological studies as described [11]. Overlapping digital low magnification (~10,000 x) images of the entire glomerular profiles were obtained using a JEOL CX100 electron microscope for estimation of podocyte volume as described below. High magnification (~30,000 x) images were obtained according a systematic uniform random sampling protocol for estimation of fraction of the volume (Vv) of podocyte cytoplasm occupied by GL3 inclusions [Vv(Inc/PC)], and podocyte average foot process width as previously described [11]. All stereological estimates were done by masked observers.

### Semi-quantitative Scoring of GL3 Inclusion Accumulation in Podocytes and Foot Process Widening

The extent of podocyte GL3 accumulation was scored on a scale of 0.0 to 4.0 according to the ISGFN methods [14] on semi-thin toluidine blue sections by a renal pathologist (K.S) masked to the order of biopsies and the study design. Foot process widening was also examined and scored semi-quantitatively by K.S as published elsewhere [8,16]. At least 3 electron micrographs at each of the 2000, 4000, and 6000 magnifications were examined. A score of “0” = no foot identified process effacement; “(+)” = foot process effacement in short segments; and “+” = foot process effacement in longer segments.

### Estimation of Podocyte Volume and Absolute Volume of GL3 Inclusions per Podocyte

Average volume of podocyte nuclei was estimated using the point-sampled intercept method (PSI) [19] with slight modification to reduce the volume-weighted property of this method. PSI is a design-based unbiased stereological approach to estimate volume-weighted shape and size independent average volume of arbitrary particles based on their random profiles [19]. It involves identification of random points (sampling points) inside the particles of interest, performing intercept measurements along random lines that pass through the sampling points and estimating the average volume of the particles based on the measured intercepts as described by Gundersen and Jensen and outlined below. The average particle volume obtained by this method, similar to any other volume estimator with sampling limited to two dimensional sections (e.g. Weibel-Gomez method [20] is volume-weighted, meaning that since larger particles are more likely to be sampled on a plane section, they are more likely to be included in the measurements, resulting in an over-estimation of the average volume compared to number weighted methods where all particles are sampled with similar probability regardless of their size.

In the originally described PSI method identification of random points in particles (here, nuclei) is satisfied by sectioning the nuclei in random directions and superimposing a point grid on the nuclear profiles. Only the nuclei hit by at least one superimposed point are included in the estimation [19]. Due to the spherical shape of the glomeruli and the complex structure of the glomerular capillaries, the podocyte nuclear profiles observable on a glomerular profile can be considered random sections through podocyte nuclei. However, limiting the intercept measurements to nuclei that are hit by a point grid, and thereby enhancing the chance of excluding smaller nuclei, leads to increasing the volume weighted property of this method. Moreover, in the original PSI method, one intercept is measured per sampling point. Therefore, more intercept measurements are included from the larger nuclei, even more enhancing the volume weighted property of this method.

**Fig 2:**
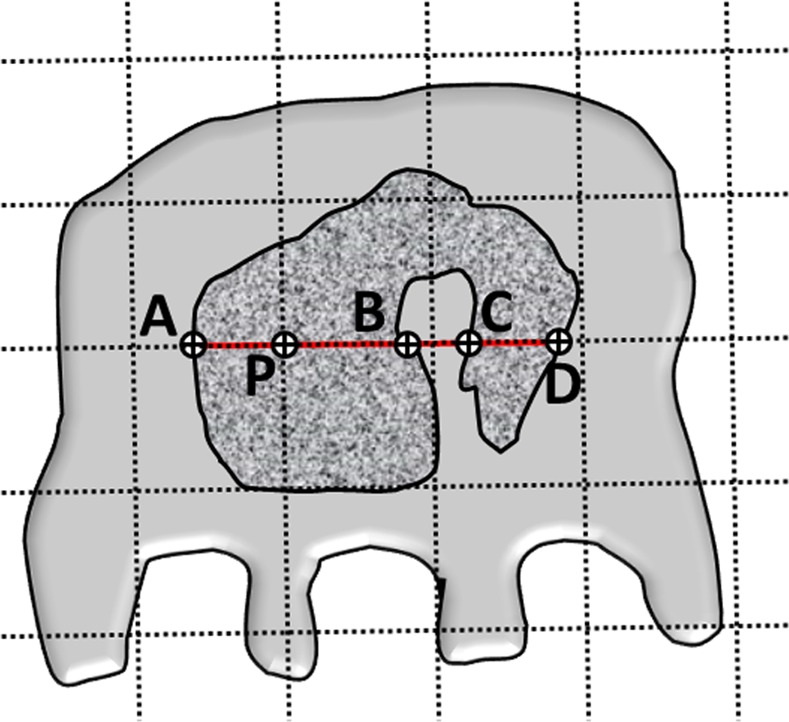
Intercept measurements for the point-sampled intercept method for a podocyte nucleus with concavity. Dashed lines represent the sampling grid superimposed on podocyte nuclei. One of the cross-points of this grid falling on the nuclear profile (here "P") is randomly selected(i.e., sampling point). The red line represents the random direction line, passing through the sampling point "P" along which the intercepts are measured. The intercepts include $l_{0,0}^{3}=AB$, $l_{0,i-}^{3}=PC$, and $l_{0,i+}^{3}=PD$.

In order to make the PSI less-volume weighted, we modified the above procedure by including all visible nuclear profiles in measurements and identifying only one random point per nucleus. For the latter, a point grid was superimposed on each nucleus and a random point was selected using a random number generator. Assuming that podocyte nuclei are positioned in random directions in the glomeruli, a horizontal and a vertical line were passed though the sampling point as random direction lines. If all nuclei are convex, an unbiased estimate of their average volume ($\bar{V}$) will be $\bar{V}=\frac{\pi }{3}\bar{l_{0}^{3}}$, where $\bar{l_{0}^{3}}$ is the average of the 3^rd^ power of the lengths of intercept of the random line and nuclear membrane passing through the sampling point. If the nuclear shape includes concavities, which is not uncommon for podocytes, the random direction line may create more than one intercept by passing through the convex areas. In such circumstance, an unbiased estimator of the average nuclear volume will be $\bar{V}=\frac{\pi }{3}(\bar{l_{0,0}^{3}}+2\bar{l_{0,e}^{3}})$, where *l*_0,0_, is the distance between the immediate intercepts of the random line with the nuclear membrane on both sides of the sampling point (Fig 2). $l_{0,e}^{3}=\sum_{i=1}^{k}l_{0,i}^{3}$ .or sum of $l_{0,i}^{3}$ over all the extra intercepts not containing the sample point, while $l_{0,i}^{3}=l_{0,i+}^{3}-l_{0,i-}^{3}$, where $l_{0,i+}^{3}$ and $l_{0,i-}^{3}$ are the longest and the shortest distances between the sampling point and intercepts of the random direction line and nuclear membrane (Fig 2).

Following estimation of average volume of podocyte nuclei, volume fraction of podocyte nuclei per podocyte [Vv(PCN/PC)] was estimated using point counting. Then, the average volume of podocytes (${\bar{V}}_{PC}$) was calculated:

V¯PC=V¯PCN.VV(PCNPC)

The absolute volume of GL3 inclusions per podocyte [V(Inc/PC)] was then calculated:

V(IncPC)=V¯PC.VV(IncPC)

### Exocytosis of GL3 Inclusions

Extrusion (exocytosis) of GL3 inclusions into the urinary space was initially studied by observation and identified as presence of inclusions that were partially intra- and partially extracellular, protruding into the urinary space. Then, the number of extrusions was normalized by the number of podocyte nuclei present in the same glomerular profile.

### Statistical Analyses

Statistica 8.0 (Statsoft, Inc.) was used. Parametric or non-parametric tests were used based on the variable characteristics and distribution. Data are presented as mean ± SD, except where indicated. Comparison of baseline and post-ERT variables was done using paired student's t-test or Wilcoxon matched pairs test. Comparison of variables in Fabry patients and normal controls was done using Student's t-test or Kolmogorov-Smirnov test. Relationships between variables were evaluated using Pearson correlation. p≤0.05 was considered statistically significant.

## Results

### Demographic and Clinical Characteristics

Baseline age was 31 [18–46] years (median [range]), urine protein/creatinine ratio was 0.2 [0.1–1.6] (median [range]) g/g, serum creatinine was 0.8 ± 0.1 mg/dL (mean ± SD) (Table 1).

**Table 1:** Clinical characteristic at baseline and follow up after 11–12 months of enzyme replacement therapy.

Case	Age, Baseline (years)	GLA Mutation	SCr/eGFR, Baseline	SCr/eGFR, Follow up	UPCR, Baseline	UPCR, Follow up
1	18	M267R	0.87/122	0.92/114	0.25	<0.18
2	23	893INSG	0.90/111	NA	0.10	NA
3	25	†	0.70/146	0.70/146	0.40	no proteinuria*
4	37	P259L	0.80/116	0.80/116	0.10	no proteinuria*
5	37	r.[195_546dup352;800_801ins217GenBank X14448:g.10293_10509, 195_546dup352]+[0]	0.70/135	0.80/116	1.62	0.45
6	46	†	0.60/154	0.50/190	0.21	no proteinuria*

Abbreviations: BL = baseline; FU = follow up; SCr = serum creatinine in mg/dl; eGFR = estimated GFR in ml/min/1.73m^2^ by MDRD formula.[18]. UPCR = urine protein/creatinine ratio in g/g (normal value <0.3); NA = not available

† Diagnosis established by low serum α-galactosidase A activity (<1.5 nmol/hr/ml which was the detection limit), no mutation detected.

* Semi-quantitative urine dipstick.

### Podocyte GL3 Content was Substantially Reduced within one Year of ERT

**Fig 3:**
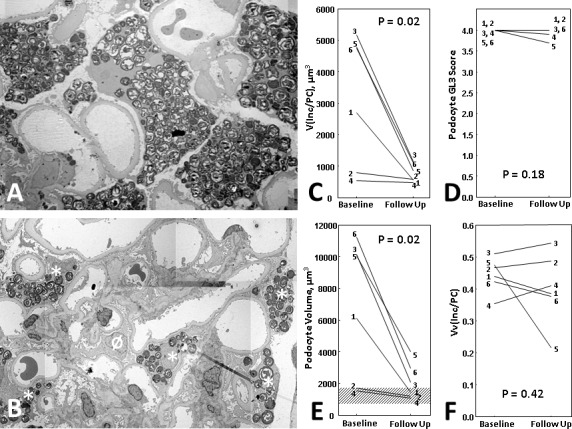
**(A)** A representative glomerulus from an 18 year old male (Case #1, see Table 1 for patient's characteristics) with Fabry disease at baseline (ERT-naïve). Podocytes (PC) are remarkably enlarged with abundant GL3 inclusions (Inc); **(B)** A representative glomerulus from the same patient after 12 months of ERT (1 mg/kg/EOH agalsidase-beta) shows smaller podocytes. The majority of podocytes still showed many GL3 inclusions (asterisks). However, occasional podocytes showed no GL3 inclusions (Ø); **(C)**Total volume of GL3 inclusions per podocytes V(Inc/PC) was reduced after 11–12 months of ERT (p = 0.02); **(D)** Podocyte GL3 score (semiquantitative [14]) did not reduce significantly after 11–12 months (follow-up) of ERT (p = 0.18); **(E)** Podocyte volume significantly decreased after 11–12 months of ERT compared to baseline (p = 0.02). The dashed area shows the range of podocyte volume in biopsies from 5 healthy kidney donor normal controls. The difference between podocyte volume in Fabry patients after 11–12 months of ERT and these healthy controls was not statistically significant; **(F)** Podocyte GL3 inclusion volume fraction [Vv(Inc/PC)] did not change significantly after 11–12 months of ERT (p = 0.42). The numbers written by each line in C-F represents case numbers according to Table 1. Panels C-F show the average values of presented parameters in each biopsy.

Most podocyte GL3 inclusions did not completely clear after 11–12 months of ERT; however, some podocytes appeared substantially less packed by GL3 inclusions in the post-ERT biopsies (Fig 3A and 3B). Average V(Inc/PC) was reduced (average 73%) following ERT (Fig 3C). Absolute volume of GL3 inclusions/podocyte [V(Inc/PC)] decreased in all patients, substantially in four and minimally in two. In contrast, while all biopsies had baseline podocyte GL3 ISGFN scores of 4.0, GL3 score was unchanged in 3 while in 3 the change ranged from 0.1 to 0.7 with an average score reduction of 0.1 from baseline (not statistically significant) (Fig 3D). Among the 223 podocyte profiles with observable nuclei on glomerular profiles in baseline biopsies, only 2 small podocytes contained no GL3 inclusions corresponding to 0 [0–6.3]% (median [range]) podocytes/biopsy. In contrast, among the 314 podocytes studied at follow-up, 12 [5–27]% podocytes/biopsy had no observable GL3 inclusions (p = 0.008). There was no relationship between %podocytes without inclusions and any clinical or structural parameters.

### Reduction of Podocyte GL3 Following ERT was Closely Paralleled by Reduced Podocyte Volume

Podocytes at baseline appeared larger than at follow-up (Fig 3A and 3B). Unbiased morphometry confirmed, that parallel to the reduction in V(Inc/PC), mean podocyte volume at follow-up was reduced by an average of 63% (Fig 3E). There was a strong correlation between the reduction in podocyte volume and the reduction in V(Inc/PC) (r = 0.88, p = 0.01), indicating proportional shrinkage of podocyte cytoplasm with the decrease in V(Inc/PC). Podocyte volume after ERT remained numerically above the values from 5 normal controls, but this was not statistically significant (Fig 3E). In contrast to the marked reduction in V(Inc/PC), Vv(Inc/PC) did not change significantly (Fig 3F).

### Exocytosis of GL3 Inclusions

**Fig 4:**
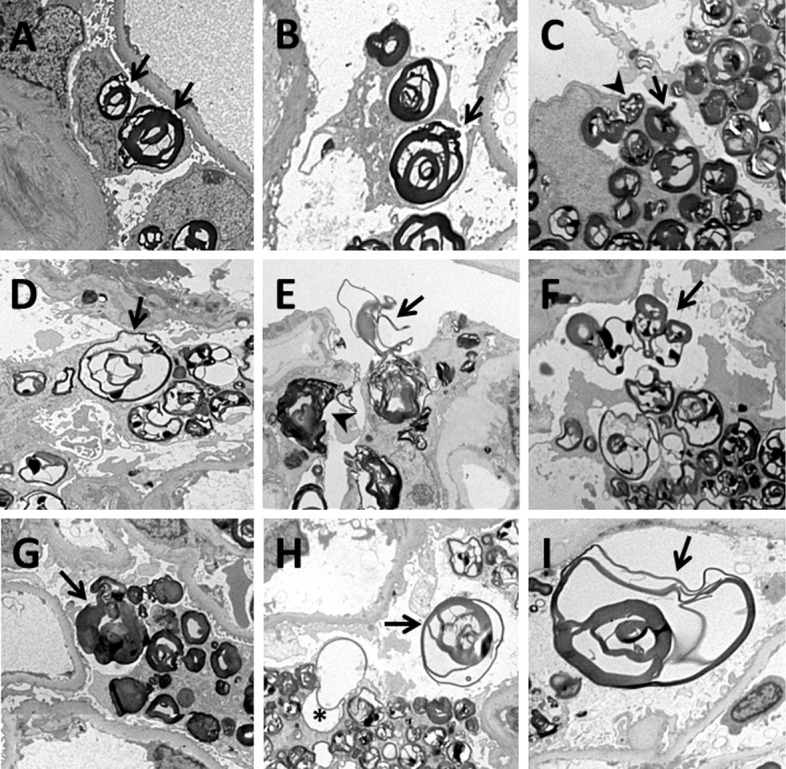
Various stages of GL3 inclusion exocytosis in podocytes. **(A)** Fusion of GL3 inclusions or their surrounding membranes with the cell membrane (arrows); **(B)** The space surrounding an intracellular GL3 inclusion is connected to the urinary space through an orifice (arrow); **(C)** A portion of a GL3 inclusion is squeezed into the urinary space through an orifice (arrow). An adjacent GL3 inclusion is partially extruded from the podocyte while preserving its round shape (arrowhead): **(D)** A GL3 inclusions extruding from a podocyte shows partial unfolding of its multilamellar structure (arrow); **(E)** Unfolding of the multilamellar structure of a GL3 inclusion while being squeezed out of a podocyte through an orifice (arrow); **(F)** Extruded GL3 inclusions in the urinary space (arrow); **(G)** A large round GL3 inclusion almost completed its extrusion from the podocyte (arrow); **(H)** An empty round space in a podocyte with partial protrusion into the urinary space, reflecting recent exocytosis (asterisk) and a nearby extracellular GL3 inclusion (arrow); **(I)** A large and partially unfolded GL3 inclusion in the urinary space (arrow).

Although intra-lysosomal digestion is a likely mechanism of GL3 reduction following ERT, we observed that podocytes, consistent with exocytosis, regularly extruded GL3 inclusions into the urinary space, (Fig 4A–4I). Many intracytoplasmic inclusions were within round spaces, presumably lysosomes. The confining membrane of some of these round spaces was focally fused with podocyte cell membranes (Fig 4A and 4D). Rarely, these round spaces close to the apical membrane of podocytes were connected to the urinary space through a small orifice (Fig 4B). While most of the inclusions extruding from podocytes retained their round shape, some seemed squeezed into thinner shapes while leaving podocytes (Fig 4C). Also, the multilamellar structure of the inclusions unfolded into thin membranes during this process (Fig 4E). Partial unfolding of multilamellar inclusions was also observed in some of the intracellular inclusions (Fig 4D). Corresponding to GL3 inclusion exocytosis, empty intracytoplasmic vacuolar spaces were sometimes seen close to the apical podocyte membranes. Extracellular inclusions (Fig 4H) in the urinary space, some undergoing partial unfolding, were frequently observed (Fig 4H and 4I).

We counted 0.33 ± 0.09 GL3 extrusions/podocyte at baseline and 0.24 ± 0.7 extrusions /podocyte at follow-up (statistically not significant). However, the number of GL3 extrusions/podocyte at baseline and after ERT strongly correlated with the total volume of GL3 inclusions/podocyte at baseline (r = 0.90, p = 0.02) and follow-up (r = 0.97, p = 0.001), respectively. Importantly, podocyte total GL3 inclusion volume reduction from baseline to follow-up correlated with the numbers of GL3 extrusions/podocyte at baseline (r = 0.87, p = 0.02) and follow-up (r = 0.85, p = 0.03), suggesting a relationship between podocyte GL3 inclusion exocytosis and GL3 clearance. The number of GL3 inclusion extrusions/podocyte at baseline and follow-up was also correlated (r = 0.81, p = 0.05).

### Foot Process Width Following ERT

**Fig 5:**
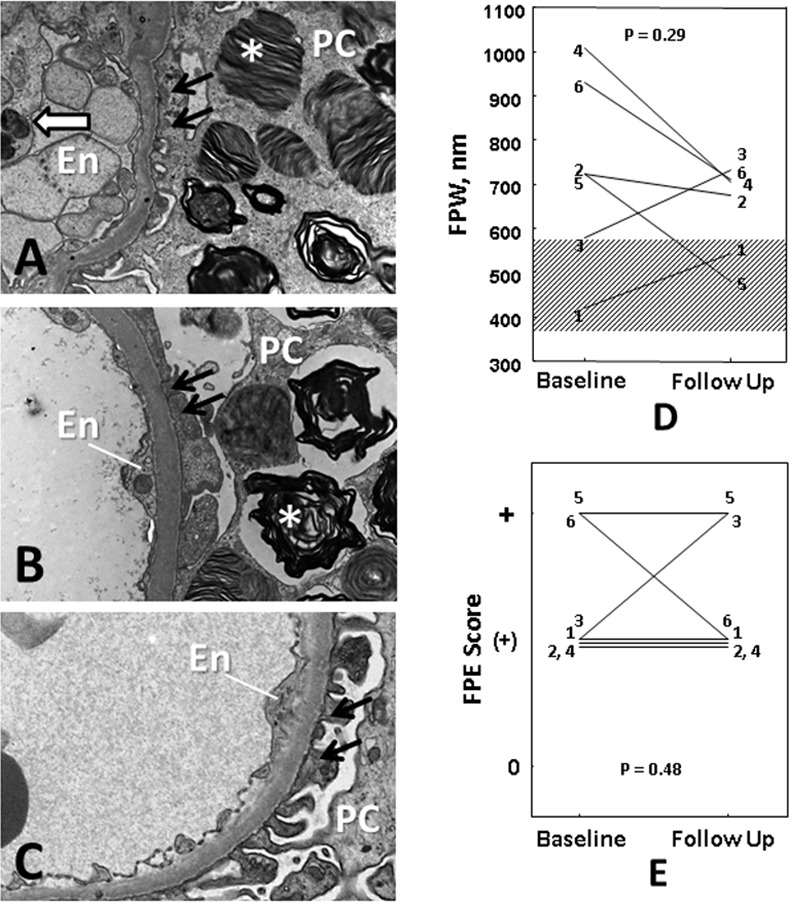
**(A)** Foot process effacement(black arrows) in a biopsy from a Fabry patient at baseline (ERT-naïve). Asterisk marks a GL-3 inclusion in a podocyte (PC). White arrow shows GL-3 inclusions in an endothelial cell (En). **(B)** A biopsy after 11–12 months of ERT from the same patient still shows areas of glomerular basement membranes with foot process effacement (black arrows). Note that podocytes contain GL-3 inclusions (asterisk), while the endothelial cells are cleared from inclusions. **(C)** Intact foot processes (black arrows) from a normal control biopsy. **(D)** Foot process width (FPW) changes estimated by unbiased morphometry. Although FPW was numerically reduced from baseline to follow up in 4/6 cases, the difference was not statistically significant (p = 0.29). The dashed area shows the range of FPW in biopsies from 9 healthy kidney donor normal controls. FPW in baseline and follow up biopsies from Fabry patients were significantly greater than normal controls (p = 0.002 and p = 0.0006, respectively). **(E)** Foot process effacement (FPE) semi-quantitative scores did not change significantly from baseline to follow up. FPE scoring was based on Tøndel et al. [16], where a score of “0” = no foot identified process effacement; “(+)” = foot process effacement in short segments; and “+” = foot process effacement in longer segments.

**Fig 6:**
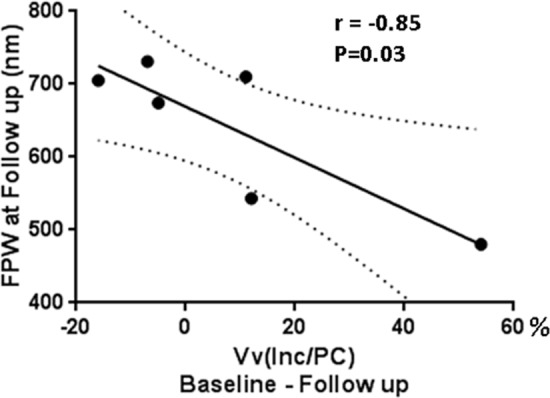
Correlation between volume fraction of GL-3 inclusions per podocyte [Vv(Inc/PC)] % change from baseline to follow up (11–12 months post ERT) and foot process width (FPW) at follow up.

Segmental foot process effacement was present in all biopsies at baseline and following ERT (Fig 5A–5C). FPW decreased on average by 12% following ERT, however, this was not statistically significant (Fig 5D). FPW after ERT was still 48% greater than in 9 normal controls (p = 0.0006, Fig 5D). Foot process effacement score did not change significantly from baseline (Fig 5E). There were no statistically significant correlationbetween decrease in V(Inc/PC) and FPW over the 11–12 months of ERT. However, the reduction in Vv(Inc/PC) from baseline to follow-up was strongly inversely correlated with the FPW after ERT (r = -0.85; p = 0.03; Fig 6). There was, however, no significant correlation between foot process effacement scores after ERT and the reduction in Vv(Inc/PC).

## Discussion

Agalsidase beta [Fabrazyme^®^ (Genzyme, a Sanofi Company)], at 1 mg/kg/every other week (EOH) for 11–12 months in previously ERT-naïve Fabry disease patients uniformly decreased the total per podocyte GL3 burden. This benefit, detected using systematic unbiased electron microscopic sampling and validated morphometric methods, was not regularly seen using semi-quantitative methods in studies that included these same [3] or longer term [4] follow-up in adults with Fabry disease. Thus, after 11 months of ERT 1mg/kg/EOH, using a 0 to 4+ light microscopic semiquantitative GL3 scoring system in plastic embedded sections, there was a reduction in podocyte scoring in only 18% of 17 patients. [3] Five of the 6 patients in the current report were, in fact, derived from this agalsidase beta study. Perhaps this discrepancy should not be surprising since the fraction of the podocyte cytoplasm occupied by GL3 [Vv(Inc/PC)] was unchanged in the follow-up biopsies in the present study, this primarily what is estimated by semi-quantitative subjective scoring. [14] The decreased per podocyte GL3 burden reported here was associated with a uniform parallel decrease in mean podocyte cytoplasmic volume, *i*.*e*., change in a parameter difficult to discern by scoring methods. Our finding that an experienced nephropathologist was unable to detect a significant change in podocyte GL3 using a published scoring system [14] supports this hypothesis. In fact, it was because this ‘reference-trap’ effect was anticipated, that a novel morphometric method to detect changes in podocyte size was developed and applied here. Importantly, increased podocyte volume was described in other pathologic conditions and was related to podocyte stress, apoptosis and loss. [21,22,23]

We also described podocyte extrusion (exocytosis) of GL3 inclusions into the urinary space and direct correlations between this and podocyte GL3 content and clearance with ERT. Although observed in our limited number of cases, this suggests that GL3 inclusion exocytosis represents a previously unrecognized but important mechanism of podocyte GL3 loss. In fact, podocytes are terminally differentiated cells with little capability to replenish their loss by cell division. [10] Thus, the ability of these cells to clear accumulated GL3 by mechanisms independent of cell division is potentially very important and worthy of further study as means of enhancing this process may provide new treatment options. We previously demonstrated a relationship between GL3 inclusion density and podocyte injury [11]. Thus GL3 inclusion exocytosis may be vital for podocyte protection. Although lysosomes are considered terminal degradative organelles, physiologic excretion of lysosomal contents occurs in multiple cells, including hepatocytes, platelets, and macrophages [24,25,26]. Regulated lysosomal exocytosis has also been described in fibroblasts and epithelial cells [27,28], and in cell membrane repair [29], neuronal regeneration [30,31], and extraction of transition metals from cells [32,33]. Regulated lysosomal exocytosis appears to be Ca^2+^ dependent [34]. Impairment of endolysosomal trafficking, and thereby, degradation of lysosomal contents through autophagy has been described in other lysosomal storage diseases, including juvenile neuronal ceroid lipofuscinosis, Pompe and Niemann Pick type C disease, multiple sulfatase deficiency and mucopolysaccharidosis type IIIA [35,36,37,38,39]. As suggested by the current study, once lysosomal degradation is impaired in lysosomal storage diseases, lysosome exocytosis may become more prominent. Therefore, better understanding of this phenomenon may lead to novel treatments for such diseases. Activation of lysosomal exocytosis by the transcription factor EB (TFEB) promoted cellular clearance in cultured cells from various lysosomal storage disease animal and human models, but Fabry disease was not tested [40]. It is important to explore similar approaches *in-vitro* and *in-vivo* in Fabry disease. Podocyte albumin exocytosis probably occurs in nephrotic rats [41], but to our knowledge, direct extrusion of particulate constructs from podocytes into the urinary space has not been previously appreciated. Since podocyte GL3 inclusions increase over time, exocytosis *per se*, is probably insufficient to prevent progressive podocyte damage. Nevertheless, the relationship between podocyte GL3 inclusion reduction following ERT and exocytosis suggests ERT-induced enhancement of exocytosis as an alternative mechanism of ERT action.

Another novel finding in relation to podocyte GL3 inclusion extrusion was the unfolding of the multilamellar structure of the inclusions, this reminiscent of unfolding of multilamellar surfactant inclusions upon leaving type II alveolar cells [42]. It will be interesting to explore if mechanisms involved in the physiological exocytosis of surfactant from alveolar cells relate to podocyte GL3 exocytosis. [43].

Also noteworthy was the regular finding of podocytes with no GL3 inclusions in the post-ERT biopsies,while these were very rare at baseline. Whether this was related to the reduced podocyte GL3 content and random sectioning through cytoplasmic regions without inclusions requires confirmation using 3-dimensional approaches. However, the absence of a relationship between Vv(Inc/PC) and %podocytes without GL3 inclusions at follow-up does not support this possibility. Alternatively, this could reflect regeneration of podocytes that, during ERT, did not accumulate GL3 inclusions, a possibility that could be difficult to prove. It will be important to examine if, with longer-term ERT, podocyte GL3 content keeps reducing and the number of podocytes without GL3 inclusions increases.

Increase in FPW is an indicator of podocyte stress. Although FPW did not correlate with proteinuria in some conditions,[44,45] marked increases in FPW well beyond what we have observed in Fabry disease have been associated with idiopathic focal segmental sclerosis (FSGS) vs. patients with minimal change nephrotic syndrome [45]. However, we have previously described direct correlations between FPW and urinary protein excretion in diabetic nephropathy [46] and in young persons with Fabry disease [47]. In the present study, It is unclear as to why FPW was not significantly decreased post- ERT by either unbiased morphometry or scoring. One possibility is that subtle decreases in FPW would require larger numbers of patients or longer treatment duration to detect due to sampling variabilities that could make small differences in paired biopsies difficult to detect. Nonetheless. This would be especially true if changes in FPW were more segmental, i.e., less evenly distributed throughout the glomerulus than, for example, in diabetic nephropathy. Nonetheless, FPW at follow-up estimated by morphometry but not by semiquantitative scoring, was strongly inversely correlated with the decrease in podocyte GL3 content from baseline to follow up. These findings, consistent with early reduction in podocyte stress by ERT, require confirmation by additional studies. Although there are no studies of scoring of podocyte effacement after ~1 year of ERT, a longer term study found no change in effacement score in 5 of 7 patients that also had no changes in podocyte GL3 as estimated by scoring. [8] As noted above, we previously demonstrated strong correlations between FPW and age, Vv(Inc/PC) and urine protein/creatinine ratio in young Fabry patients [11], this consistent with the progressive nature of Fabry nephropathy. In contrast, such relationships were undetectable by scoring of podocyte effacement [16]. Thus, as for podocyte GL3 content, systematic unbiased sampling and morphometric measurements are more sensitive than subjective scoring as indicators of change in another important Fabry disease podocyte parameter, FPW. It is possible that refinements in the scoring of FPW would better approximate morphometrically measured FPW and this is worth pursuing.

There are several reasons for focus on the podocyte as a treatment target in Fabry disease. First, as mentioned, Fabry nephropathy risk increases with age as does Vv(Inc/PC) while endothelial and mesangial cell GL3 fractional volumes do not. [11] Also Vv(Inc/PC) correlates with urinary protein levels [11] and proteinuria is a strong risk predictor for GFR loss in Fabry nephropathy. [6] Finally, endothelial and mesangial cell GL3 inclusions clear completely after brief periods of ERT whereas podocyte GL3 inclusions are much more persistent. [3] Despite endothelial and mesangial cell clearance there are residual risks for Fabry patients on ERT. Since these risks are related to the severity of the proteinuria when ERT is initiated [6], the relatively poor clearance of GL3 from podocytes may be of substantial clinical significance. Thus, effects on podocyte GL3 content may be better than effects on other glomerular cell types as a marker of Fabry disease treatment adequacy and residual risk.

Therapies designed as additions to ongoing ERT will not have endothelial and mesangial cells as treatment readouts as they will be cleared of GL3 inclusions at baseline. [3] In contrast, the incomplete effects of ERT on podocyte GL3 provides opportunities for testing the value of new treatments to supplement ERT. Moreover, using the morphometric methods developed here, such trials could be shorter and require fewer participants than if based on subjective scoring or clinical progression. Finally, the methods outlined here would also be applicable to other important cell types that are relatively resistant to ERT, especially arterial smooth muscle cells and cardiac myocytes.[3,48,49]

There may be concern that these morphometric methods are more time consuming and expensive than scoring systems, require special training to perform and are thus not available as routine procedures. While true, these facts should be considered in light of the very high health risks faced by Fabry disease patients, the costs of lifelong ERT approaching $200,000/year, the need for more effective therapies for many patients, and the relatively small Fabry disease patient population. In our view, it is reasonable to consider establishing a few reference laboratories using uniform validated methods for evaluating renal biopsy and other vital tissues affected by Fabry disease. Another approach would be to use quantitative morphometric methods as the ‘gold standard’ in order to refine scoring methods that could be more broadly implemented.

These studies have some limitations. Greater numbers of paired biopsies in ERT treated Fabry disease patients could allow elucidation of predictors of response such as baseline disease severity, mutation, age, and treatment dose and duration. Also we did not study females. The finding of GL3 negative podocytes in treated males and mosaciasm with GL3 negative podocytes in pre-ERT females [50] would require estimation of numbers of GL3 negative podocytes at baseline and follow-up as well as the separate measurement of podocyte volume and Vv(Inc/PC) in affected cells in such patients. Moreover, such approaches would be required in studies in males of therapies added to ongoing ERT.

In summary, morphometric studies regularly detected reductions in the absolute volume of GL3 inclusions per podocyte after 11–12 months of ERT at 1 mg/kg/EOH in males with Fabry disease. This was in association with reductions in podocyte size rather than the proportion of GL3 filled podocyte cytoplasm. Some podocytes after ERT had no observable GL3. FPW reduction after ERT correlated with the reduction in podocyte GL3. Extrusion of GL3 inclusions was noted and correlated with the decline in podocyte GL3. This study, which depended on novel methods to estimate changes in mean podocyte volume, requires extension to larger numbers of Fabry disease patients in order to better understand the variables associated with Fabry disease renal injury and treatment response and to design trials based on the podocyte as a key treatment outcome.
